# Gains of 20q11.21 in human pluripotent stem cells: Insights from cancer research

**DOI:** 10.1016/j.stemcr.2023.11.013

**Published:** 2023-12-28

**Authors:** Nuša Krivec, Manjusha S. Ghosh, Claudia Spits

**Affiliations:** 1 Research Group Reproduction and Genetics, Faculty of Medicine and Pharmacy, Vrije Universiteit Brussel, Brussels, Laarbeeklaan 103, 1090 Brussels, Belgium

**Keywords:** human pluripotent stem cells, clinical translation, cancer, 20q11.21, genetic integrity

## Abstract

The genetic abnormalities observed in hPSC cultures worldwide have been suggested to pose an important hurdle in their safe use in regenerative medicine due to the possibility of oncogenic transformation by mutant cells in the patient posttransplantation. One of the best-characterized genetic lesions in hPSCs is the gain of 20q11.21, found in 20% of hPSC lines worldwide, and strikingly, also amplified in 20% of human cancers. In this review, we have curated the existing knowledge on the incidence of this mutation in hPSCs and cancer, explored the significance of chromosome 20q11.21 amplification in cancer progression, and reviewed the oncogenic role of the genes in the smallest common region of gain, to shed light on the significance of this mutation in hPSC-based cell therapy. Lastly, we discuss the state-of-the-art strategies devised to detect aneuploidies in hPSC cultures, avoid genetic changes *in vitro* cultures of hPSCs, and strategies to eliminate genetically abnormal cells from culture.

## Teaser

This review examines the current knowledge on the prevalence and implications of the gain of 20q11.21 in cancer, with the aim of providing insight into the potential oncogenesis of hPSC-derived cells posttransplantation. We review the role of the genes in the minimal region of gain in oncogenic transformation and the influence of the mutation on cancer progression, and discuss methods to detect, eliminate, and minimize its appearance.

## Introduction

With over 50 ongoing or recently concluded Phase I/II clinical trials (www.clinicaltrials.gov), human pluripotent stem cells (hPSC)–derived cells are taking their first steps into the clinic (Kobold et al., 2020), showing promising results in the treatment of previously incurable diseases, such as age-related macular degeneration (Liu et al., 2018b; Schwartz et al., 2012; 2015; Song et al., 2015), type 1 diabetes (Ramzy et al., 2021), and Parkinson disease (Doi et al., 2020; Kirkeby et al., 2017; Piao et al., 2021), as reviewed by Nguyen et al. (2018) and Yamanaka (2020).

Despite this potential, the transfer of hPSCs from lab to clinic still faces several challenges, including ethical concerns regarding the use of human embryonic stem cells (hESCs) (Rosner et al., 2014), finding cost-effective methods to bypass allogenicity, and establishing optimal differentiation methods, reviewed by Nguyen et al. (2018). In addition, there are growing concerns about the susceptibility of hPSC cultures to acquire mutations during *in vitro* growth (Andrews et al., 2022; Keller and Spits, 2021). These genetic lesions range from single base to full chromosome aberrations (Nguyen et al., 2013), including epigenetic changes (Bar and Benvenisty, 2019). The most commonly reported abnormalities are gains of (parts of) chromosomes 1, 12, 17, 20, and X (International Stem Cell Initiative et al., 2011), and dominant-negative TP53 mutations (Avior et al., 2021; Merkle et al., 2017, 2022). These genetic changes start, at the single-cell level, as random events (Jacobs et al., 2014, 2016; Keller et al., 2019), but a subset provides the cells with a selective advantage that allows them to be fixed in the culture through cell competition. The fact that the same aberrations are common in both hESCs and hiPSCs suggests that they play a role in adaptive evolution in culture (International Stem Cell Initiative et al., 2011; Taapken et al., 2011). However, the mechanisms by which these abnormal cells outcompete their neighbors has been established for a few of them (Avery et al., 2013; Ben-David et al., 2014; Markouli et al., 2019; Merkle et al., 2017; Nguyen et al., 2014; Price et al., 2021). The impact of genetic mutations on hPSC differentiation is still not well understood, with only a few studies systematically analyzing multiple lines carrying the same aberration. Overall, all of the reports coincide in that genetically unbalanced cells show abnormalities in lineage specification (Ben-David et al., 2014; Herszfeld et al., 2006; Jo et al., 2020; Markouli et al., 2019; Werbowetski-Ogilvie et al., 2009; Yamamoto et al., 2022).

Next to impairing differentiation and cell maturation, another important reason for concern is that these abnormalities bear a striking resemblance to the mutations found in cancer (Andrews et al., 2022; Baker et al., 2007; Keller and Spits, 2021; Merkle et al., 2017; Oliveira et al., 2014; Simonson et al., 2015). There is little insight as to whether and how these mutations could affect the oncogenic capacity of transplanted hPSC-derived cells. Most of the research on this topic has concentrated on addressing the risk of tumor formation by residual undifferentiated cells or by highly proliferative progenitor cells in the differentiated cell product (Allison et al., 2018; Andrews et al., 2022; Ben-David and Benvenisty, 2011). With this concern in mind, much work has been devoted to developing methods to generate highly pure cell populations and to avoid the presence of any undifferentiated hPSC in the final product (Ben-David et al., 2013a; Choo et al., 2008; Kuang et al., 2017; Matsumoto et al., 2016; Shiraki et al., 2014), as reviewed by Keller et al. (2018), Masuda et al. (2014), and Yamanaka (2020). Recent work has shown that digital droplet PCR can detect as few as 0.001% of residual undifferentiated cells (Piao et al., 2021), which is crucial because the contamination of transplanted differentiated cells with 0.03% of undifferentiated cells can result in a teratoma in the recipient (Wang et al., 2020a).

Conversely, the genetic abnormalities seen in hPSC could be regarded as a “first hit” in a cancerous transformation. Transplanted hPSC-derived cells that carry these genetic abnormalities will as such not be cancerous, but may have a greater chance of undergoing oncogenic transformation by requiring fewer additional genetic hits. Although this notion is particularly compelling in the case of the p53 mutations recurrently identified in hPSCs (Avior et al., 2021; Merkle et al., 2017, 2022), it can hold equally true for aneuploidy. For example, human glioblastoma frequently starts with cells acquiring gains of chromosome 7 and losses on chromosome 10 (Körber et al., 2019). If transplanted hPSC-derived neural cells already carried one of these aberrations, further spontaneous mutagenesis could result in neoplastic transformation in the recipient.

One of the challenges is that because aneuploid cells are in many cases still able to correctly differentiate, they would not show as residual undifferentiated or poorly differentiated cells, and would as such pass undetected in the final differentiated cell population. The current strategy to minimize the risks associated with transplanting genetically abnormal hPSC-derived cells is to subject hPSC cultures and products to genetic screening before their use in patients. However, the standard methods for genetic screening may not detect abnormalities present as a low-grade mosaic in the hPSC culture, in which only a fraction of the cells carries an abnormality. Abnormal cell populations are common in hPSC cultures, with as many as 20% of cells of cultures that test normal by conventional karyotyping actually carrying a variety of genetic imbalances (Jacobs et al., 2014; Keller et al., 2019). If any of these abnormalities functions as a first hit in the oncogenic process, then the transplantation of such a cell product could have, in the worst-case scenario, tumor-initiating capacities.

In this review, we provide an overview of the parallels between cancer cells and hPSCs in regard to the gain of 20q11.21, one of the most common and best-characterized genetic lesions seen in hPSCs (International Stem Cell Initiative et al., 2011; Merkle et al., 2022), and one that has been implicated in a wide range of cancers. We focus on the functional significance of the genes present in this region and their possible role in priming hPSC-derived cells for malignant transformation, with the ultimate aim of providing insight into the potential risks of transplanting hPSC-derived products contaminated with cells carrying this mutation.

## Gains of chromosome 20 in hPSC

**Figure 1:**
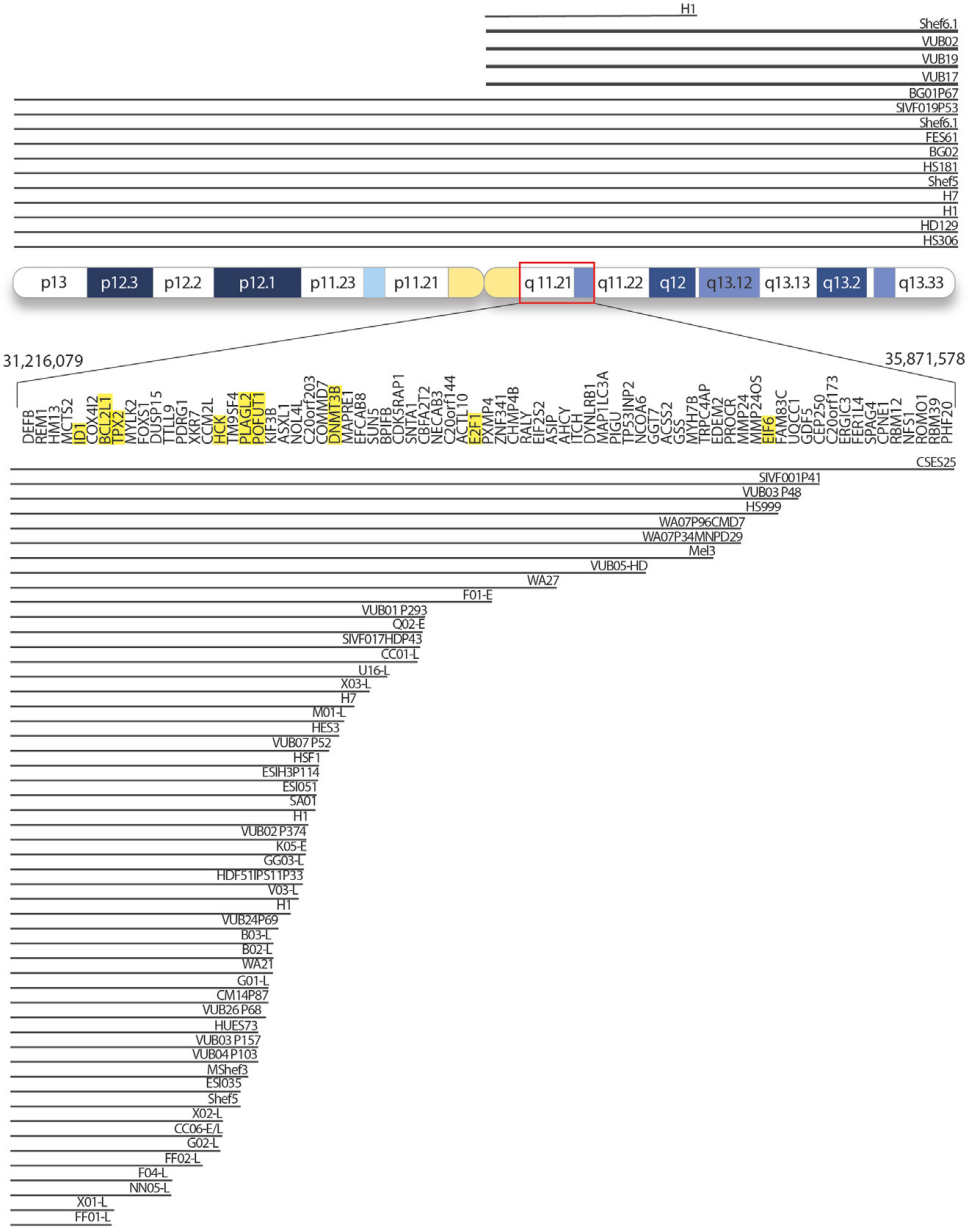
Overview of the hPSC lines reported to carry a gain of (part of) chromosome 20 Each black line represents a separate cell line, and the length of the line indicates the size of the gain. The lines at the top of the ideogram represent the entire chromosome 20, with thin lines indicating trisomy and bold lines representing a gain through an isochromosome 20q. The black lines at the bottom represent cell lines with gains that start in the 20q11.21 region. The largest duplication begins at position 31,216,079 and ends at 35,871,578 based on build hg38, spanning 4.6 Mb. Information on the references and exact breakpoints of each cell line can be found in Table S1. The genes marked in yellow are discussed in detail in this review.

Trisomy or gain of part of chromosome 20 is found in over 20% of hPSC lines worldwide (Assou et al., 2020; Avery et al., 2013; Baker et al., 2007; Catalina et al., 2008; International Stem Cell Initiative et al., 2011; Laurent et al., 2011; Maitra et al., 2005; Merkle et al., 2022; Mitalipova et al., 2005; Närvä et al., 2010; Nguyen et al., 2014; Rosler et al., 2004; Spits et al., 2008; Taapken et al., 2011) (Figure 1). At least 11 hPSC lines with trisomy 20 have been reported in the literature, 4 with an isochromosome 20q and 53 cell lines with a gain that always starts at 20q11.21 and spans varying lengths of the 20q arm. The minimal amplicon is located in the 20q11.21 region; it is 0.56 Mb long and contains 13 genes. The mutation is likely the result of replication fork stalling and collapse, followed by microhomology-mediated break-induced replication, and is facilitated by repetitive sequences. The proximal breakpoint of the gain of 20q is always in the pericentromeric microsatellite region, and the distal breakpoints are located close to Alu sequences, with a common (GGAAT)n sequence identified in the breakpoints of different cell lines (Halliwell et al., 2021; Merkle et al., 2022). Once the cell has acquired the gain, it rapidly takes over the culture. The selective advantage of the gain of 20q11.21 is thought to be mediated by the increased expression of the antiapoptotic protein Bcl-xL coded by the gene *BCL2L1* located in the minimal region of gain. Bcl-xL inhibits the mitochondrial apoptosis pathway and confers cells’ better survival upon culture-related cellular stress, such as passaging (Avery et al., 2013; Nguyen et al., 2014). Conversely, the mutation has also been reported to lead to further genetic instability. The antiapoptotic effect of the gain of *BCL2L1* has been suggested to render the cells insensitive to mitotic stress (Zhang et al., 2019), whereas the concurrent amplification of *TPX2* causes deregulation of the microtubule network organization, which can lead to chromosome misalignment and abnormal mitosis (Jeong et al., 2023). Also, the high expression of *TPX2* causes high Aurora A activity and YAP1 stabilization, which further induces the transcription of *BCL2L1* (Kim et al., 2023).

The impact of the mutation on the cells is not limited to the undifferentiated state, but extends to the ability of the cells to correctly and efficiently differentiate (Jo et al., 2020; Markouli et al., 2019; Vitillo et al., 2023; Werbowetski-Ogilvie et al., 2009). Lines with a gain of 20q11.21, as well as cells transgenically overexpressing Bcl-xL, show alterations in the transcriptome, affecting, among others, the transforming growth factor-β (TGF-β)/SMAD signaling. These, in turn, result in a reduced capacity to differentiate into neuroectoderm, whereas the mesoderm specification remains unaffected (Jo et al., 2020; Markouli et al., 2019). hPSCs with a gain of 20q11.21 have also been shown to produce vascularized teratomas and to exhibit a reduced potential to form hematopoietic lineages from embryonic bodies (Pridgeon et al., 2023; Werbowetski-Ogilvie et al., 2009). Recently, hPSCs carrying an isochromosome 20q have been reported to fail to undergo correct germ layer specification upon spontaneous differentiation and rather undergo apoptosis or specify to extraembryonic and amnion tissue, avoiding the epiblast developmental trajectory (Vitillo et al., 2023).

## Gains of chromosome 20 in cancer

**Figure 2:**
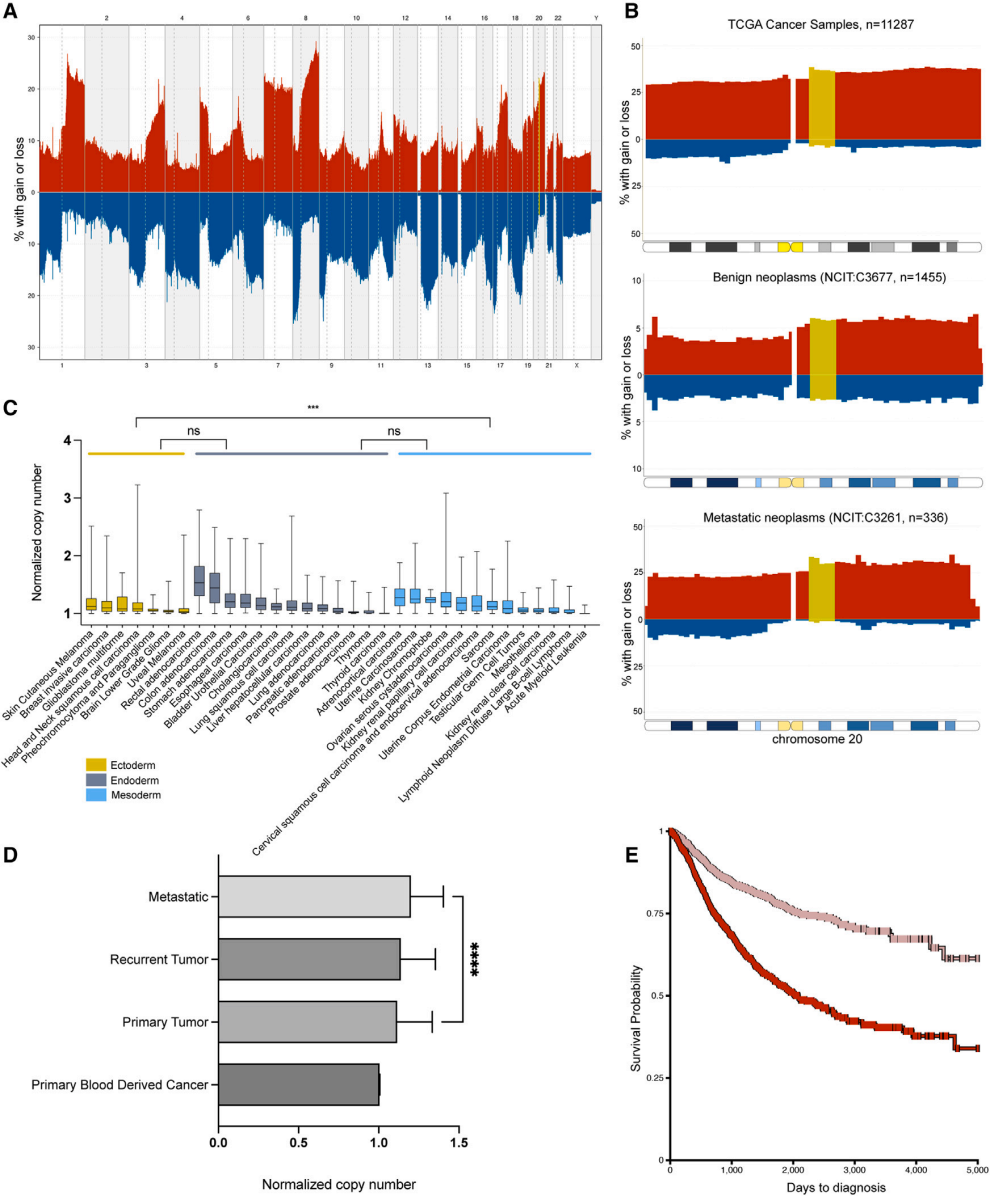
Copy-number alterations of human chromosome 20q11.21 in cancers (A) Aggregated copy-number variation (CNV) data of 117,587 neoplasms (NCIT: C3262) from the Progenetix database (Huang et al., 2021) were plotted using R library pgxRpi. The percentage of samples with aberrations (red, gain; blue, loss) for the whole chromosome are indicated on the y axis. Chromosomal regions are depicted on the x axis; the minimal region of interest at chr20:31216079-35871578 is marked in moss green. NCIT, National Cancer Institute Thesaurus. (B) Top to bottom: Aggregated CNV data of 11,287 TCGA cancer samples, 336 metastatic neoplasms (NCIT: C3261), and 1,455 benign neoplasms (NCIT: C3677) from the Progenetix database (Huang et al., 2021), respectively, were plotted using R library pgxRpi. The percentage of samples with aberrations (red, gain; blue, loss) for the whole chromosome are indicated on the y axis. Chromosomal regions are depicted on the x axis; the minimal region of interest at chr20:31216079–35871578 is marked in moss green. (C) Amplification of chromosome 20q11.21 (31216079–35871578) found across various tumor types categorized according to the germ layer to which the cell of origin of the tumor belongs. Box and whiskers plot for normalized copy-number gain (tumor/normal) of chromosome 20q11.21 (31216079–35871578) in respective cancers as indicated. The box extends from the 25th to the 75th percentile, whiskers indicate the smalles and largest values, the median is indicated as a line in the box. Data extracted from TCGA-PANCAN database using the University of California, Santa Cruz (UCSC) Xena online platform (Goldman et al., 2020), skin cutaneous melanoma (n = 403), breast invasive carcinoma (n = 848), glioblastoma multiforme (n = 535), head and neck squamous cell carcinoma (n = 414), pheochromocytoma and paraganglioma (n = 154), brain lower-grade glioma (n = 451), uveal melanoma (n = 52), rectal adenocarcinoma (n = 150), colon adenocarcinoma (n = 367), bladder urothelial carcinoma (n = 358), cholangiocarcinoma (n = 29), esophageal carcinoma (n = 160), liver hepatocellular carcinoma (n = 278), lung adenocarcinoma (n = 360), lung squamous cell carcinoma (n = 430), pancreatic adenocarcinoma (n = 154), prostate adenocarcinoma (n = 419), stomach adenocarcinoma (n = 338), thymoma (n = 53), thyroid carcinoma (n = 342), adrenocortical carcinoma (n = 74), cervical squamous cell carcinoma and endocervical adenocarcinoma (n = 228), lymphoid neoplasm diffuse large B cell lymphoma (n = 24), kidney chromophobe (n = 60), kidney renal clear cell carcinoma (n = 417), kidney renal papillary cell carcinoma (n = 168), AML (n = 107), mesothelioma (n = 73), ovarian serous cystadenocarcinoma (n = 521), sarcoma (n = 199), testicular germ cell tumors (n = 97), uterine corpus endometrial carcinoma (n = 297), and uterine carcinosarcoma (n = 50). The Dunn’s test p value is indicated between groups of 3 germ layers: ectoderm (n = 2,857), yellow; endoderm (n = 3,438), gray; and mesoderm (n = 2,315), blue; ns, not significant; ∗∗∗ p=0.0006. Significant differences in amplifications were found across various tumor types within groups, ectoderm (Kruskal-Wallis test, p < 0.0001), endoderm (Kruskal-Wallis test, p < 0.0001), and mesoderm (Kruskal-Wallis test, p < 0.0001), not indicated in the figure." (D) 20q11.21 has a significantly increased copy number in metastatic samples. Normalized copy numbers (tumor/normal) are depicted on the x axis (median with interquartile range) for primary blood-derived cancers (n = 118); primary tumor (n = 8,610), recurrent tumor (n = 53) and metastatic (n = 332) samples. Copy number of metastatic samples is significantly higher compared to the primary tumors (Mann-Whitney *U* test, p < 0.0001). No significant difference was observed between primary tumor and recurrent tumors (Mann-Whitney *U* test, p = 0.8441). Data were extracted from TCGA database using the UCSC Xena online platform (Goldman et al., 2020). (E) Patients carrying amplifications of 20q11.21 have poor disease-specific survival. Kaplan-Meier survival analysis curve for patients carrying balanced (log[tumor/normal] < 0.03600, n = 1,993, pink) and amplified (log[tumor/normal] > 0.3054, n = 1,932, red) loci, p = 0.00. Primary tumors carrying aberration values of log(tumor/normal) ≥ 0 at 20q11.21 (31216079–35871578) loci were used to select for samples. Kaplan-Meier curve was made using the UCSC Xena online platform (Goldman et al., 2020). The x axis depicts time in days to diagnosis and the y axis depicts probability of survival. Source data for the entire figure can be found in Table S2.

Gain of one or multiple copies of chromosome 20q11.21 is found in 20% of all benign and malignant neoplasms (n = 117,587; Figure 2A). The frequency with which the gain appears varies across neoplasm type. In pancreatic cancers, 60% of samples carried gain of region 20q11q13.2 (Kitoh et al., 2005), and 52% of oral squamous cell carcinoma samples had a minimal region of gain of 20q11.21q13.33 (Vincent-Chong et al., 2013). In invasive cervical cancer, over 50% of samples carry gains of the entire chromosome, the full long arm or focal amplifications of 20q11.2 and 20q13.13 (Scotto et al., 2008). Also, in colorectal cancer, two focal subregions of amplification were found—20q11.2q12 and 20q13 (Tsafrir et al., 2006). Although the deletion of chromosome 20q is common in blood cancers *such as acute myeloid leukemia (*AML), a report found targeted amplifications of 20q11.21 in 18% of the samples (Mackinnon et al., 2010). It is worth noting that gain of 20q commonly appears concomitant to additional genetic aberrations, frequently as part of a complex karyotype. Also, some abnormalities appear to more frequently associate with gains of 20q in specific cancers. For instance, in 19% of ovarian cancers, chromosome 20q11 gain cooccurs with gain of 19q12 (Gorringe et al., 2010); in gastric cancer, gain of 20q11.21 or 20q13.12 is significantly associated with gain of 8q24 (Jin et al., 2015).

To have a comprehensive overview of the incidence and nature of aberrations on chromosome 20, we assessed the incidence of chromosome 20 copy-number abnormalities across 11,287 The Cancer Genome Atlas (TCGA) cancer samples (Figure 2B). Gains appeared more frequently than losses, with 20q11.21 showing across all cancer types an average amplification frequency of 38.39% at chromosomal position 20:311–321 Mb, 37% at 20:321–331 Mb, 36.8% at 20:341–351 Mb, and 36% at 20:351–361 Mb. Losses were rare, having a frequency of 3%–4% in the 20:311–361–Mb region. Interestingly, the incidence of gains of 20q11.21 is quite lower in benign (5%–6%, n = 1,455) than metastatic (30%–33%, n = 336) lesions, which suggests that tumor cells carrying gains of 20q have increased propensity toward malignant transformation (Figure 2B). We next looked at the amplification levels of 20q11.21 over various TCGA cancer types (Figure 2C) to explore whether there is a particular cell lineage preferentially acquiring gains at 20q11.21. We categorized the cancers on the basis of the germ layer to which their “cell of origin” belongs. Despite the significant heterogeneity in the frequency of the amplification within cancers from the same layer—ectoderm (Kruskal-Wallis test, p < 0.0001), endoderm (Kruskal-Wallis test, p < 0.0001), and mesoderm (Kruskal-Wallis test, p < 0.0001)—all cancers of ectodermal origin (n = 2,857) have significantly lower levels of amplification in 20q11.21 compared to mesodermal (n = 2,315) cancers (pairwise nonparametric Dunn’s test, p = 0.0006). No significant difference was observed between pairs with endoderm (n = 3,438) cancers. Colon and rectal adenocarcinomas had the highest levels of amplification, in line with the literature, and blood-derived cancers such as AML have the lowest incidence. Taken together, this suggests that the amplification of 20q11.21 has a pronounced context-dependent effect in the development and progression of cancer.

The role of 20q11.21 in promoting the metastatic progression of cancer has been investigated in several studies. A systematic review of clear-cell renal cell carcinoma showed that gain of 20q 11.21 is one of the most frequent gains, with a prevalence rate of 50%, and that it was significantly increased in metastatic samples as compared to primary tumors (Bui et al., 2022). Gains in the 20q11.21q13.33 region are specifically involved in liver metastasis of colorectal cancer (Yamamoto et al., 2010). In a study on paired analysis of oral tongue squamous cell carcinoma primary and lymph node metastasis samples, gain of 20q11.21 was found to facilitate metastasis (Morita et al., 2016). Furthermore, gain of 20q significantly correlated with lymph node metastasis status of gastric adenocarcinomas, where in lymph node–positive gastric adenocarcinomas, 85% had a gain of 20q, as opposed to 14.6% cases without the 20q gain (Buffart et al., 2009). Also, in esophageal squamous cell carcinoma (Tada et al., 2000), gains of 20q were observed in 9 of 24 tumors with lymph node metastases compared to 0 of 12 tumors without lymph node metastases. To further explore the role of 20q11.21 in metastatic progression, we plotted the 20q11.21 copy-number data of samples, including primary, recurrent, and metastatic tumors and blood cancers from the TCGA Cancer (Pancreatic Cancer Action Network [PANCAN]) database (Figure 2D). We selected for samples with balanced copy number or gains of 20q11.21, excluding samples with losses (20% of samples, 2,211 out of 10,873). Metastasis samples (n = 332) have a significant increase (Mann-Whitney *U* test, p < 0.0001) in the copy number of 20q11.21 as compared to the primary tumors (n = 8,610), suggesting a role for the gain of 20q11.21 in aiding cancer progression. This same database allowed us to look at the survival rates of patients with a cancer carrying gain of 20q11.21. We extracted the disease-specific survival through ∼13 years of cancer patients carrying a gain (upper quartile at log[tumor/normal] > 0.3054) or balanced copy number (lower quartile log[tumor/normal] < 0.03600) of this region in their primary tumors and found that patients with high levels of amplification on chromosome 20q11.21 (minimal region spanning positions 31216079 to 35871578 of chromosome 20) have poorer disease-specific survival probability (Figure 2E; p = 0, primary tumors with losses were not included). This is irrespective of the cancer type and in line with reports that find that increased copy numbers of 20q11.21 and of chromosome 20 are linked to poor prognosis and survival, for instance, in colorectal and breast cancers (Nakopoulou et al., 2002; Voutsadakis, 2021).

**Figure 3:**
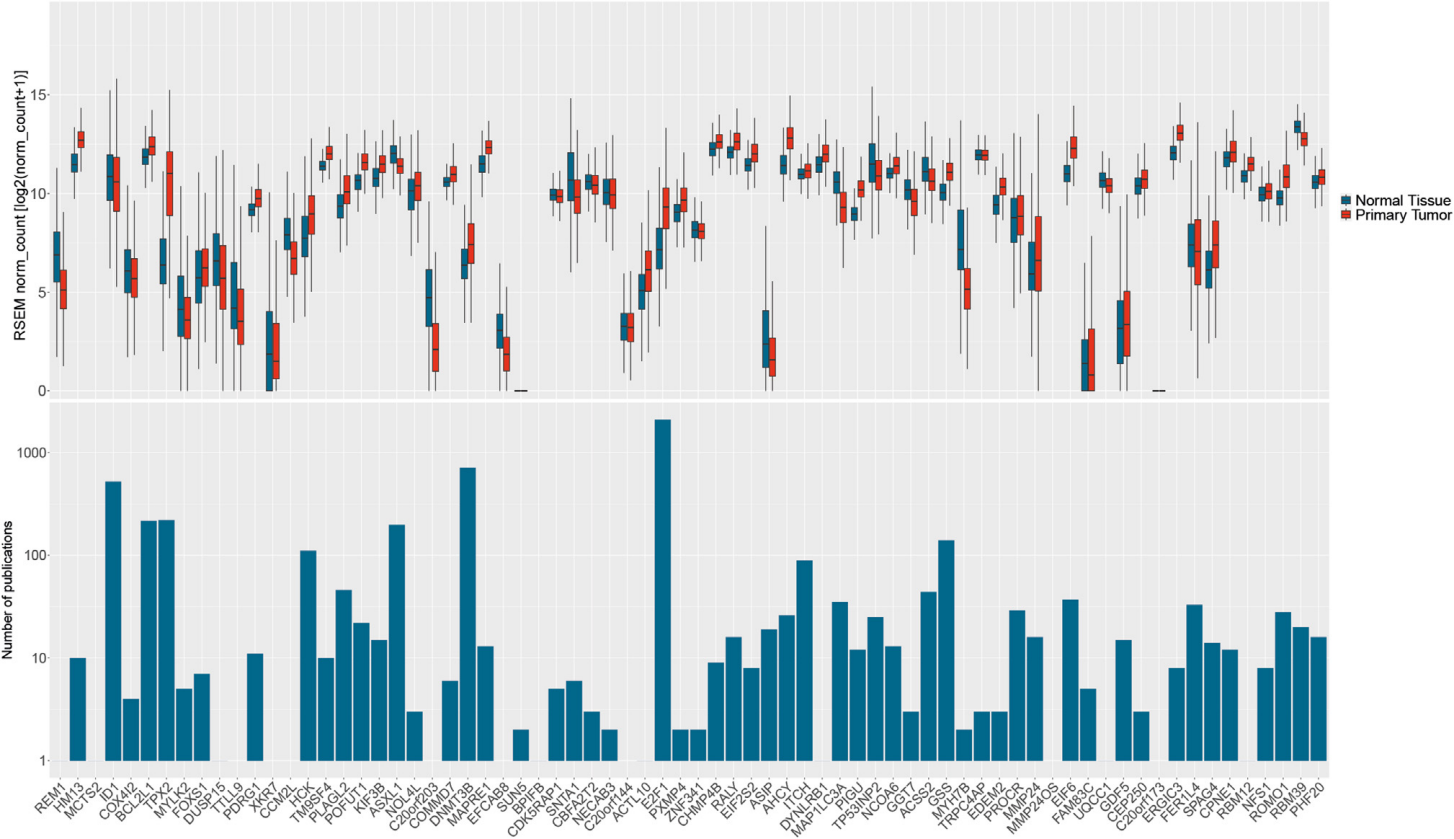
Gene expression and number of publications related to tumorigenesis per gene on chromosome 20q11.21 The criteria for the most relevant genes to study in this review were gene expression data in primary tumors and number of publications in the PubMed database. Gene expression in primary tumor tissues from TCGA database was compared to gene expression in normal tissue from GTEx in the UCSC Xena online platform (Goldman et al., 2020). In the box and whisker plot, the box comprises from 25^th^ to 75^th^ percentiles, the whiskers extend from the hinge to the largest and smallest values no further than 1.5-times the inter-quartile range from the hinge, and the line indicates the median. Genes with higher expression were considered together with the number and quality of publications in PubMed. The search in PubMed database was filtered with the following search terms: (“gene name” [Title/Abstract]) AND ((cancer[Title/Abstract]) OR (tumor[Title/Abstract])). Results are shown in the bottom graph in the logarithmic scale. The genes that correspond to the criteria are *ID1*, *BCL2L1*, *TPX2*, *HCK*, *PLAG2*, *POFUT1*, *DNMT3B*, *E2F1*, and *E2F6*. Source data for the figure can be found in Table S3.

Considering the above, we further reviewed the mechanisms by which the increased copy number of 20q11.21 plays a role in oncogenesis. We selected a subset of the genes present in the common region of gain for further review, based on whether the genes are differentially expressed in cancers as compared to somatic tissues, and on the number of articles on PubMed discussing the function of these genes in cancer (Figure 3). We narrowed down the list to *ID1*, *BCL2L1*, *TPX2*, *HCK*, *PLAG2*, *POFUT1*, *DNMT3B*, *E2F1*, and *E2F6*. It is worth noting that none of these genes are cataloged as oncogenes according to the Catalogue of Somatic Mutations in Cancer Cancer Gene Census (https://cancer.sanger.ac.uk/census), indicating that despite this evidence, their roles in cancer are still in need of further research.

## Genes in the minimal region of gain of 20q11.21 with a role in cancer

### ID1

*ID1* belongs to the family of ID proteins that regulate proliferation, differentiation, and cell senescence by binding to basic-helix-loop-helix transcription factors and regulating the G1 to S phase transition (Hara et al., 1994). *ID1* overexpression has been found in various cancers, and it has been identified as a prognostic predictor of lymph node metastasis and reduced survival in patients with penile cancer (Hu et al., 2019; Zekan et al., 2021). In breast cancer, *ID1* is involved in lung metastasis in a mouse model and in patients (Minn et al., 2005). *ID1* overexpression is found in hyperplastic, neoplastic, and malignant thyroid cancer tissues (Kebebew et al., 2004). In salivary adenoid cystic, oral squamous cell, and prostate carcinoma, *ID1* expression correlates with tumor progression and shorter disease-free survival (Hu et al., 2019; Ponz-Sarvisé et al., 2014; Zhao et al., 2013). *ID1* has also been found to be implicated in the shorter survival of AML patients (Zhou et al., 2015).

### BCL2L1

*BCL2L1* encodes two proteins of the BCL-2 family: proapoptotic Bcl-xS and antiapoptotic Bcl-xL (Dou et al., 2021). Several cancers present dysregulated splicing and overexpression of the antiapoptotic isoform Bcl-xL, including solid and hematological malignancies (Dou et al., 2021; Ma et al., 2010; Morales-Martínez and Vega, 2022). Bcl-xL expression levels in tumor samples correlate with higher tumor grades and shorter relapse-free survival in glioma (Ita et al., 2022), chondrosarcoma (de Jong et al., 2018), tongue carcinoma (Zhang et al., 2014), triple-negative breast cancer (Skov et al., 2022), and melanoma (Gabellini et al., 2018). In melanoma, Bcl-xL overexpression leads to the increased secretion of proinflammatory chemokine interleukin-8 (IL-8), which leads to aggressive tumors (Gabellini et al., 2018). In breast cancer, Bcl-xL is suggested to play a role in invasion and migration (Keitel et al., 2014), and in the highly invasive basal B subtype of triple-negative breast cancer, the combined overexpression of Bcl-xL and Aurora A has been found to promote metastasis (Skov et al., 2022). In pancreatic neuroendocrine tumors, the role of Bcl-xL in metastasis is independent of its antiapoptotic activity (Choi et al., 2016) and instead could be due to an increase in TGF-β signaling. This interestingly links to the deregulated TGF-β signaling observed in hESCs with a gain of 20q11.21 and their abnormal response to neuroectoderm differentiation (Markouli et al., 2019).

### TPX2

Targeting protein for Xklp2 (*TPX2*) is a microtubule-binding protein that contributes to the spindle assembly and function during cell division (Wadsworth, 2015). *TPX2* has been found to be amplified and overexpressed in a wide variety of tumors, which in turn has been associated with cancer progression, metastasis, recurrence, and a poor prognosis in, for instance, breast cancer (Jiang et al., 2019), esophageal cancer (Sui et al., 2019), endometrial cancer (Wang et al., 2022), hepatocellular carcinoma (Huang et al., 2019), and colorectal carcinoma (Neumayer et al., 2014). *TPX2* can promote the activation of AKT and the phosphatidylinositol 3-kinase (PI3K)/AKT signal transduction pathways, and the downregulation of *TPX2* inhibits cell proliferation and promotes cell apoptosis in breast cancer and hepatocellular carcinoma by regulating the expression of proteins such as BCL-2, c-Myc, cyclin D1, p21, caspase-3, and BAX (Chen et al., 2018; Huang et al., 2019).

### HCK

*HCK* is a member of the Src family of tyrosine kinases and is primarily expressed in hematopoietic cells (Luo et al., 2023). *HCK* is overexpressed in many types of leukemia and solid malignancies (Poh et al., 2015). In colorectal tumorigenesis, *HCK* acts as a driver of cell transformation into adenoma (Zheng et al., 2022). Excessive HCK activation is also corelated with enhanced cell proliferation, enhanced secretion of growth factors and pro-inflammatory cytokines, and chemoresistance (Poh et al., 2015). In breast cancer, it regulates immune response signaling pathways and cell growth through the epithelial-to-mesenchymal transition, the PI3K/AKT signaling pathway, and focal adhesions (Zhu et al., 2020). *HCK* overexpression enhances osteosarcoma tumorigenesis via the MEK/ERK pathway *in vitro* (Liu et al., 2021) and enhances *in vitro* cell viability, proliferation, and migration in glioblastoma cell lines by the TGF-β-induced epithelial-to-mesenchymal transition process (Wang et al., 2020b).

### PLAGL2

The pleomorphic adenoma-like gene 2 (*PLAGL2*) is a transcription factor that is highly expressed in cancerous tissues compared to adjacent nontumor tissues (Hensen et al., 2002; Keck et al., 2023; Lin et al., 2023). The expression levels of *PLAGL2* correlate to tumor size, metastasis status, and clinical stage in bladder urothelial carcinoma (Qu et al., 2018), colorectal adenocarcinoma (Wang et al., 2017), and gastric cancer (Wu et al., 2020). In colorectal cancer, the overexpression of *PLAGL2* induces the epithelial-to-mesenchymal transition, a key process in metastatic tumor progression, by activating the Wnt/β-catenin signaling pathway (Wang et al., 2017). *PLAGL2* has also been found to regulate Wnt/β-catenin signaling in malignant gliomas and to promote self-renewal in neural progenitors (Zheng et al., 2010). *PLAGL2* is co-regulated with *POFUT1* by a bidirectional promoter, and the two genes synergistically promote tumorigenesis in colorectal cancer by maintaining stemness and cell-cycle deregulation (Li et al., 2019).

### POFUT1

Protein *O*-fucosyltransferase 1 (POFUT1) is an enzyme that adds *O*-fucose to various proteins with epidermal growth factor–like repeats, including Notch (Shi and Stanley, 2003). Several studies have suggested that POFUT1 plays a role in tumor progression. *POFUT1* expression is increased in colorectal adenomas, which are precursors to colorectal cancer (Komor et al., 2020). *POFUT1* overexpression activates Notch1 signaling, which promotes adenoma-to-carcinoma progression (Komor et al., 2020). In breast cancer, high *POFUT1* expression has been observed in infiltrating ductal carcinomas as compared to adjacent normal tissue (Wan et al., 2017). Tumors with high *POFUT1* levels have a higher histological grade and advanced stage and increased risk of lymph node metastasis in breast, gastric, and hepatocellular carcinomas (Dong et al., 2017; Ma et al., 2016; Wan et al., 2017). In addition, the overexpression of *POFUT1* correlates with decreased disease-free survival in hepatocellular carcinoma and glioblastomas (Li et al., 2021; Ma et al., 2016).

### DNMT3B

DNMT3B is a *de novo* DNA methyltransferase that plays a role in maintaining methylation patterns in the human genome (Yanagisawa et al., 2002). The overexpression of *DNMT3B* is common to many cancers and has been associated with poor clinical outcomes in hepatocellular carcinoma (Lai et al., 2019), oral cancer (Chen et al., 2014), esophageal carcinoma (Chen et al., 2012), and triple-negative breast cancer (So et al., 2022). Its overexpression is correlated with the expression of IL-6 in hepatocellular carcinoma and oral cancer (Chen et al., 2014; Lai et al., 2019). IL-6 activates signal transducer and activator of transcription 3 signaling in the hepatocytes, leading to increased proliferation and tumor formation (He and Karin, 2011). Transformed cells overexpressing *DNMT3B* have been shown to have an increased ability to undergo the epithelial-to-mesenchymal transition, which contributes to their metastatic abilities (So et al., 2022).

### E2F1

E2F transcription factor 1 (*E2F1*) plays a central role in the retinoblastoma and p53 tumor-suppressor pathways (Nakajima et al., 2023; Palacios et al., 2008). The overexpression of *E2F1* is a sign of transformation and progression in various types of cancer, such as melanoma (Nelson et al., 2006), bladder cancer (Mun et al., 2020), and breast and lung carcinomas (Tsantoulis and Gorgoulis, 2005). *E2F1* is highly upregulated in late-stage tumors and promotes cancer invasion and metastasis in prostate cancer (Chun et al., 2020) and colon cancer (Fang et al., 2020). *E2F1* confers a selective growth advantage to premalignant or transformed cells in the prostate (Chun et al., 2020) and is also regulated by c-Myc, which tightly controls proliferation (O’Donnell et al., 2005). Interestingly, E2F1 can enhance *NANOG* expression by binding its promotor region, promoting stemness in breast cancer cells (Lu et al., 2018).

### EIF6

Eukaryotic translation initiation factor 6 (EIF6) plays a crucial role in translation regulation, ribosome synthesis, and cell-fate determination (Benelli et al., 2012). It is a downstream effector of Notch1 signaling, which leads to increased cell migration and invasive phenotype (Benelli et al., 2012). The overexpression of EIF6 correlates with the poor prognosis of patients in many cancers, including gall bladder cancer (Golob-Schwarzl et al., 2019), lung adenocarcinoma (Shen et al., 2023), esophagus adenocarcinoma (Gao et al., 2022), hepatocellular carcinoma (Sun et al., 2021), melanoma (Zhang et al., 2022), and colorectal cancer (Lin et al., 2019). *EIF6* amplification and its subsequent overexpression was found to be a driver of highly proliferative luminal breast cancers (Gatza et al., 2014). *EIF6* expression positively correlates with stemness-associated genes in lung adenocarcinoma cells (Shen et al., 2023), and knocking out *EIF6* improved prognosis in mice. EIF6 triggers mammalian target of rapamycin signaling in hepatocellular carcinoma, leading to enhanced proliferation and invasion (Sun et al., 2021). In colorectal cancer, EIF6 activates AKT-related cellular signaling to increase tumorigenesis by modulating cell proliferation, cell cycle, and apoptosis (Lin et al., 2019).

## Strategies to minimize genetically abnormal cells in culture

Considering the evidence above, it is desirable to develop strategies to avoid, reliably detect, or eliminate this abnormality in culture, and particularly in cell products destined for clinical application. Here, we discuss the strengths and limitations of current genetic screening methods, the efforts made to improve hPSC culture conditions, and innovative methods to target and eliminate specific aneuploid cells.

### Detecting low-grade mosaicism for gains of 20q11.21

An array of methods has been used for the analysis of chromosomal changes in hPSCs, including G-banding, fluorescence *in situ* hybridization (FISH), microarray-based comparative genome hybridization (aCGH) (Andrews et al., 2017), qPCR, digital droplet PCR (ddPCR) (Baker et al., 2016), whole-genome sequencing (WGS) (Keller et al., 2019), and virtual karyotyping (Ben-David et al., 2013b). They each differ in resolution, sensitivity, labor intensity, price, and sample type (Andrews et al., 2022; Keller and Spits, 2021), and their suitability depends on the context in which the cells are karyotyped. It is clear that for research purposes, the cells do not require the same level of characterization as they do for clinical translation. In the context of research, the International Society for Stem Cell Research has recently released new basic and preclinical standards for the use of hPSCs that include practical guidelines for genome characterization (https://www.isscr.org/standards).

To detect low-grade mosaicism for the gain of 20q11.21, even though G-banding provides information on the entire genome, it is unable to detect abnormalities smaller than 5 Mb, precluding many of the gains of 20q11.21, and the usually limited number of analyzed metaphases limits its ability to detect very-low-grade mosaicism (Rohani et al., 2018). aCGH could detect 5%–20% of cells carrying the gain of 20q11.21 in a bulk DNA sample (Andrews et al., 2022; Jacobs et al., 2014), and WGS can do so when more than 4% of the cells carry the abnormality (Merkle et al., 2022). Virtual karyotyping (e-karyotyping) can detect abnormalities larger than 10 Mb and mosaicism down to 30% (Ben-David et al., 2013b), which would allow it only to identify the larger 20q gains.

Targeted approaches to detect specific aneuploidy are likely more suited for the identification of very-low-grade mosaicism. FISH on interphase cells is able to detect 0.5%–5% abnormal cells with a specific aneuploidy (Baker et al., 2007). For gains of 20q11.21, there is, for instance, a commercial FISH probe set that targets the region, as well as the 1q, 12q, and 17p arms (Thermo Fisher Scientific), and multiple FISH probes exist that cover the *BCL2L1* gene located in the common region of gain (Empire Genomics). PCR-based methods such as qPCR or ddPCR are interesting alternatives that do not require cell fixation because they use DNA samples extracted from the bulk cell cultures. Although qPCR is easy to implement in any laboratory, is low cost, and has a short turnaround time, ddPCR requires highly specialized equipment and expensive materials. To use qPCR to detect gains of 20q11.21, both commercial assays (e.g., the assay Hs01892845 for *ID1* [Thermo Fisher Scientific]) and custom-designed primer sets can be used (Baker et al., 2016). ddPCR also requires target assays, such as the BioRad assay dHsaCP2506319 for *ID1*. However, the limit of mosaicism detection for the PCR methods is approximately 5%–10%, which suggests that a small but significant population of abnormal cells can remain undetected (Andrews et al., 2022; Assou et al., 2018, 2020; Baker et al., 2016; Kuroda et al., 2015). Lastly, single-cell DNA sequencing has likely the highest sensitivity in detecting low-grade mosaicism, but it is also more expensive and time-consuming; therefore, it is unlikely to be suitable for routine practice (Keller and Spits, 2021).

### Minimizing the appearance of genetically imbalanced cells in culture

Culture conditions can contribute to the maintenance of the genomic integrity of hPSCs. Factors such as fast cell proliferation (Holubcová et al., 2011; Merkle et al., 2022; Weissbein et al., 2014), growing cells in high density (Jacobs et al., 2016), prolonged time in culture (Jacobs et al., 2016; Merkle et al., 2022), passaging methods (Garitaonandia et al., 2015), medium composition (Jacobs et al., 2016; Liu et al., 2018a), oxygen level (Forsyth et al., 2006; Thompson et al., 2020; Weissbein et al., 2014), and type of extracellular matrix (Garitaonandia et al., 2015) have been studied in relation to genome instability (Molina-Ruiz et al., 2022).

hPSCs exhibit a fast proliferation and reduced cell-cycle time due to rapid progression through G1 phase driven by the atypical expression of cyclins compared to somatic cells, and perturbed DNA replication predisposes them to genomic damage. hPSC grown at a high density on mouse feeder layers are exposed to high levels of lactate that lowers the pH of the medium, resulting in replication stress, DNA damage, and increased genome instability. More frequent refreshments or sodium bicarbonate supplements to the medium mitigated this effect, suggesting that changes in medium composition could bypass the effects of the cell waste products on their genome integrity (Jacobs et al., 2016; Liu et al., 2018a). The addition of exogenous nucleosides has been shown to improve hPSC DNA replication dynamics and to reduce DNA replication stress, opening a second avenue for culture condition improvements (Halliwell et al., 2020). Another possibility for diminishing the effect of the fast proliferation rates of hPSCs would be to manipulate the cell cycle by inhibiting CDK2, a regulator of G1/S transition that is highly expressed in hPSCs. A moderate inhibition of CDK2 has been shown to reduce centrosomal amplification without inducing cell differentiation and could be used in culture medium to increase genome stability (Holubcová et al., 2011; Weissbein et al., 2014). Growing hPSCs in low oxygen conditions (5%) that mimic the environment in early embryos has been found to help maintain the cells in the pluripotent state and to drive the hPSC toward anaerobic glycolysis, leading to reduced levels of reactive oxygen species in the medium and less DNA damage (Forsyth et al., 2006; Guo et al., 2013; Kuijk et al., 2020; Li and Marbán, 2010; Thompson et al., 2020). In line with this, hPSCs exhibit a 50% reduction in mutation rate under low oxygen compared to standard conditions (Thompson et al., 2020).

Most of the mutations arise during the last cell population doubling, which means that mutations will always arise during *in vitro* propagation, but the risk of specific oncogenic mutations is low. By minimizing the time in culture, we can avoid the accumulation of mutations. Enzymatic passaging is associated with higher levels of genetic aberrations compared to the mechanical passaging (Assou et al., 2020; Bai et al., 2014; Garitaonandia et al., 2015). Enzymatic reagents cause proteolytic cleavage of receptors and proteoglycans at the cell surface, which reduces the responsiveness to growth factors and leads to stress (Badur et al., 2015).

### Selectively eliminating genetically abnormal cells

Another possible approach would be to devise strategies to selectively eliminate aneuploid stem cells in culture. Residual undifferentiated PSCs have been successfully eliminated from the differentiated cell product by various chemical methods such as small-molecule inhibitors (Ben-David et al., 2015), targeting of claudin 6 (Ben-David et al., 2013c), or the use of lentivirus to specifically kill undifferentiated cells (Ide et al., 2018). However, this strategy is not useful in this case because the hPSCs harboring a gain of 20q11.21 do not specifically remain undifferentiated. In cancer research, efforts have been made toward understanding the molecular mechanisms leading to the enhanced survival of aneuploid cells to expose any vulnerability that could be exploited to develop targeted therapies to eliminate such cells. For instance, aneuploid cells have deregulated cell-cycle processes such as longer S phase and increased DNA replication fork stalling, and they show increased expression of DNA repair genes (Garribba et al., 2023; Passerini et al., 2016; Santaguida and Amon, 2015; Santaguida et al., 2017). In these lines, two ongoing clinical trials in patients with advanced cancers (NCT05028218 and NCT03096054) use CDC7 inhibitors to target the cells’ successful DNA damage response; blocking CDC27 inhibits replication in cells, which leads to cell death (González-Garrido and Prado, 2022). In liver cancer, CDC7 inhibition has also been shown to induce senescence in cells harboring p53 mutations (Wang et al., 2019). Another study showed that aneuploid cells overcome spindle assembly checkpoint (SAC) inhibition and keep proliferating and accumulating mitotic defects. They are dependent on mitotic kinesin KIF18 for this phenotype, and targeting KIF18 sensitizes the aneuploid cells further to SAC inhibition. Thus, inhibiting KIF18 and SAC together depletes aneuploid cells and is potentially relevant for therapy (Cohen-Sharir et al., 2021).

## Conclusions

To summarize, approximately 20% of cancers carry a gain of chromosome 20q11.21. Higher levels of amplification are found primarily in solid tumors originating from mesodermal and endodermal germ layers, with colorectal cancers having the highest amplitude of gains, suggesting a context-specific effect depending on the cell type or origin of the cancer. Amplification of 20q11.21 is significantly more common in metastatic cancers, and patients carrying amplified 20q11.21 in their primary tumors have a worse survival rate as compared to those having balanced copies of this region. The review on the functions of *ID1*, *BCL2L1*, *TPX2*, *HCK*, *PLAG2*, *POFUT1*, *DNMT3B*, *E2F1*, and *E2F6* in cancer reveals a plethora of pathways by which the higher expression of these genes, as a result of their increased copy number, affects the progression of cells through different stages of malignancy.

Circling back to the starting point of this review, it is clear that the safety concerns of transplanting hPSC-derived tissue bearing aneuploid cells are well founded, especially in regard to the gain of 20q11.21. If transplanted hPSC-derived cells already carried this gain, even in a small proportion of the cells, then all of the evidence suggests that further mutagenesis could rapidly result in neoplastic transformation in the recipient. It is worth noting, however, that we lack any information on whether this will be effectively the case, and research in this direction will be crucial to realistically assess the risk of transplanting cells with this abnormality. In the meantime, it is imperative to develop robust strategies to minimize the risk of transplanting hPSC-derived cells contaminated with cells carrying this mutation, which should include targeted methods for their detection and developing approaches for the propagation and differentiation of hPSCs that minimize their appearance or are able to prune out the mutant cells. Significant efforts have been already made in this direction, but further work will be needed to establish the optimal methods to be used in a clinical setting.

## Supplemental information

Table S1. CNV breakpoints This table contains the information on the references and exact breakpoints of each cell line that are shown in Figure 1.

Table S2. CNV of 20q11.21 in human cancers This table contains the data used to make each of the panels in Figure 2.

Table S3. Genes on 20q11.21 This table contains the data used to create the panels in Figure 3.
